# Prevalence and risk factors for latent tuberculosis infection among healthcare workers in Morocco

**DOI:** 10.1371/journal.pone.0221081

**Published:** 2019-08-15

**Authors:** Ayoub Sabri, Jocelyn Quistrebert, Hicham Naji Amrani, Ahmed Abid, Adil Zegmout, Ismail Abderrhamani Ghorfi, Hicham Souhi, Abdelhalim Boucaid, Anas Benali, Rachid Abilkassem, Mohamed Kmari, Amal Hassani, Belyamani Lahcen, Samir Siah, Erwin Schurr, Stéphanie Boisson-Dupuis, Jean-Laurent Casanova, Amine Lahlou, Abdelkader Laatiris, Lhoussain Louzi, Aziz Ouarssani, Ahmed Bourazza, Aziz Aouragh, Bensghir Mustapha, Nezha Messaoudi, Aomar Agader, Aurélie Cobat, Laurent Abel, Jamila El Baghdadi

**Affiliations:** 1 Genetics Unit, Military Hospital Mohamed V, Rabat, Morocco; 2 Laboratory of Human Genetics of Infectious Diseases, Necker Branch, INSERM U1163, Paris, France; 3 Imagine Institute, Paris Descartes University, Paris, France; 4 Department of Pulmonology, Military Hospital Moulay Ismail, Meknes, Morocco; 5 Medical and Pharmacological College, Mohamed Benabdellah University, Fes, Morocco; 6 Department of Pulmonology, Military Hospital Mohammed V, Rabat, Morocco; 7 Medical and Pharmacy School of Rabat, Mohammed V University, Rabat, Morocco; 8 Department of Paediatrics, Military Hospital Mohammed V, Rabat, Morocco; 9 Emergency Department, Mohammed V Military Hospital, Rabat, Morocco; 10 Department of Restorative Plastic Surgery and Burns, Military Hospital Mohammed V, Rabat, Morocco; 11 Infectious Diseases and Immunity in Global Health Program, Research Institute of the McGill University Health Centre, Montreal, Quebec, Canada; 12 St. Giles Laboratory of Human Genetics of Infectious Diseases, Rockefeller Branch, The Rockefeller University, New York, New York, United States of America; 13 Howard Hughes Medical Institute, New York, New York, United States of America; 14 Paediatric Haematology-Immunology Unit, Necker Hospital for Sick Children, Paris, France; 15 Center of Virology and of Infectious and Tropical Diseases, Mohammed V Military Hospital, Rabat, Morocco; 16 Department of Microbiology, Military Hospital Moulay Ismail, Meknes, Morocco; 17 Department of Neurology, Military Hospital Mohammed V, Rabat, Morocco; 18 Department of Gastroenterology, Military Hospital Mohammed V, Rabat, Morocco; 19 Department of Anesthesiology and Resuscitation, Military Hospital Mohammed V, Rabat, Morocco; 20 Laboratory of Hematology and Immunohematology, Military Hospital Mohammed V, Rabat, Morocco; Agencia de Salut Publica de Barcelona, SPAIN

## Abstract

Increased prevalence of latent tuberculosis infection (LTBI) has been observed among high-risk populations such as healthcare workers (HCWs). The results may depend on the method of LTBI assessment, interferon-gamma release assay (IGRA) and/or tuberculin skin test (TST). Here, we investigated the prevalence and risk factors for LTBI assessed by both IGRAs and TST in HCWs living in Morocco, a country with intermediate tuberculosis (TB) endemicity and high BCG vaccination coverage. HCWs were recruited in two Moroccan hospitals, Rabat and Meknes. All the participants underwent testing for LTBI by both IGRA (QuantiFERON-TB Gold In-Tube, QFT-GIT) and TST. Different combinations of IGRA and TST results defined the LTBI status. Risk factors associated with LTBI were investigated using a mixed-effect logistic regression model. The prevalence of LTBI among 631 HCWs (age range 18–60 years) varied from 40.7% (95%CI 36.9–44.5%) with QFT-GIT to 52% (95%CI 48.2–56.0%) with TST using a 10 mm cut-off. The highest agreement between QFT-GIT and TST (κ = 0.50; 95%CI 0.43–0.56) was observed with the 10 mm cut-off for a positive TST. For a definition of LTBI status using a double positive result for both QFT-GIT and TST, significant associations were found with the following risk factors: being male (OR = 2.21; 95%CI 1.40–3.49; p = 0.0007), belonging to age groups 35–44 years (OR = 2.43; 95%CI 1.45–4.06; p = 0.0007) and even more 45–60 years (OR = 4.81; 95%CI 2.72–8.52; p = 7.10^−8^), having a family history of TB (OR = 6.62; 95%CI 2.59–16.94; p = 8.10^−5^), and working at a pulmonology unit (OR = 3.64; 95%CI 1.44–9.23; p = 0.006). Smoking was associated with LTBI status when defined by a positive QFT-GIT result (OR = 1.89; 95%CI 1.12–3.21; p = 0.02). A high prevalence of LTBI was observed among HCWs in two Moroccan hospitals. Male gender, increased age, family history of TB, and working at a pulmonology unit were consistent risk factors associated with LTBI.

## Introduction

Approximately one quarter of the world population is estimated to have latent tuberculosis infection (LTBI) [1]. At this stage, infected subjects do not experience clinical symptoms and are not contagious. However they have a 5–10% life-time risk of developing active tuberculosis (TB) [2], which is most pronounced during the early stage of infection [3], thereby also becoming an important source of TB contagion. LTBI screening in targeted populations is thus pivotal in order to prevent the spread of TB. Healthcare workers (HCWs) have a higher risk of contracting TB than the general population through occupational exposure [4]. Several studies have investigated the prevalence of and the risk factors for LTBI in HCWs [4–7] in countries highly endemic for TB. Reported pooled prevalences ranged from 37 to 63% in these recent meta-analyses [4–7] with a variability depending on important factors such as countries’ income, Bacillus Calmette-Guérin (BCG) vaccination coverage, and LTBI status definition.

The diagnosis of LTBI is inferred indirectly from quantitative measurements of anti-mycobacterial immunity, attesting to induction of acquired immunity to *Mycobacterium tuberculosis* [8]. The tuberculin skin test (TST) is done *in vivo* and consists of an intradermal injection of purified protein derivative (PPD) that provokes a delayed hypersensitivity reaction at the site of injection. It is a sensitive test widely used but it lacks specificity, partly due to cross-reactions with BCG, and, to a lesser extent, non-tuberculous mycobacteria [9]. Another type of tests, interferon-gamma release assays (IGRAs), are performed *in vitro* and detect the secretion of interferon-gamma (IFN-γ) by leukocytes that recognize *M*. *tuberculosis*-specific antigens. In particular, the QuantiFERON-TB Gold In-Tube test (QFT-GIT) is based on the specific *M*. *tuberculosis* antigens ESAT-6, CFP10 and TB7.7 (Qiagen, Hilden, Germany). The specificity of these tests is higher than TST and is not affected by BCG vaccination [10]. IGRA results are not fully concordant with TST findings, but they provide complementary information about infection status [11–13]. The World Health Organization recommends tailored LTBI management based on tuberculosis burden and resource availability using either the TST or IGRAs [14]. However, the performance of the two tests is not known in some specific settings. In this study, we investigated the prevalence and risk factors for LTBI assessed by both IGRAs and TST in HCWs from Morocco, a low-middle-income country with an intermediate annual incidence of TB (99/100,000 in 2017), and high BCG vaccination coverage [15–16].

## Materials and methods

### Study design and population

A cross-sectional study was conducted from March 2012 to September 2016 in two Moroccan cities, Rabat and Meknes. HCWs were recruited from two hospitals, the Military Hospital Mohammed V of Rabat and the Military Hospital Moulay Ismail of Meknes. During the four years period of enrollment, the TB incidence in Morocco was stable around 100/100,000. We included in the study all adult HCWs with an age between 18 and 60 years who accepted to participate. Exclusion criteria were ongoing pregnancy, immunosuppressive treatment, history of clinical TB disease, known chronic HIV, hepatitis B or C infections, as well as known cancer or chronic renal failure. Information on the following variables was obtained using a questionnaire: gender, age, comorbidities such as diabetes or asthma (yes/no), smoking status (yes/no), history of TB in family members (yes/no), BCG vaccination, working area and occupation. The working area variable consisted of four relevant categories: non-clinical department (such as administrative sectors or laboratories, with indirect exposure to patients), surgical department (direct exposure to patients but low risk of airborne transmission), clinical department (direct exposure to patients) and the pulmonology unit (direct exposure to high-risk patients). The occupation variable was divided into three categories: the non-medical staff (such as administrative personnel, secretaries), the paramedical staff (lab technicians and auxiliaries), and the medical staff (physicians and nurses). All HCWs declared to have been vaccinated with BCG at birth. The sample size was determined by the number of HCWs who fulfilled the inclusion criteria and responded favorably to the questionnaire. The study was approved by the ethics committee of the Faculty of Medicine of Casablanca, Morocco. Written informed consent was obtained from each participant.

### QuantiFERON-TB Gold In-Tube test (QFT-GIT) assays

QFT-GIT testing was performed on blood samples collected before the TST was carried out, in accordance with the kit manufacturer’s instructions. Briefly, 1 ml of peripheral blood was drawn directly into each of three blood collection tubes under vacuum: the nil tube or negative control, the mitogen tube containing phytohaemagglutinin or positive control and the TB specific antigen tube containing specific antigens (early secretory antigenic target 6, culture filtrate protein 10 and TB 7.7 antigen) for *M*. *tuberculosis*. The contents of each tube were mixed and the tubes were incubated at 37°C for 16 to 24 hours within 16 hours of collection. Then they were centrifuged at 2500 rpm for 15 minutes at room temperature. The supernatant was stored at -20°C. The concentration of IFN-γ in each sample was determined by QFT-GIT ELISA (enzyme-linked immunosorbent assay) kit. QFT-GIT results, calculated as TB antigen minus nil value (IU/mL), were categorized as positive (≥0.35 IU/mL, QFT+), negative (<0.35 IU/mL, QFT-) or indeterminate following the software provided by the manufacturer. To further investigate discordant results with TST, QFT-GIT results were sub-classified as strong negative (<0.2 IU/mL), weak negative (0.2–0.34 IU/mL), weak positive (0.35–0.7 IU/mL) and strong positive (>0.7 IU/mL) [17–18].

### Tuberculin skin test

The tuberculin skin test (TST) was performed by the medical staff, in accordance with routine hospital procedure. Two units of standardized purified protein derivative (PPD) solution (2 T.U Tuberculin PPD RT 23, Statens Serum Institut, Copenhagen, Denmark) were injected intradermally into the forearm of all the participants using the Mantoux method. Results were evaluated after 72 hours of the intradermal injection. The transversal diameter of the induration was measured in millimeters. We considered three different cut-offs (≥ 5, 10 and 15 mm) for a positive result (TST+) to compare with QFT-GIT results. Based on our agreement results, previous studies in similar settings [5] and published guidelines [19], the 10 mm cut-off was used to assess LTBI risk factors.

### Statistical analysis

The concordance between QFT-GIT and TST results was calculated as the number of double positive (QFT+/TST+) plus double negative (QFT-/TST-) test results divided by the total number of test results. The agreement between QFT-GIT and TST results was also calculated using Cohen’s kappa coefficient (κ) and its 95% confidence intervals (CIs). Fisher’s exact test was used to compare the proportion of QFT-GIT subcategories between concordant QFT/TST and discordant QFT/TST results. The TST induration between QFT-/TST+ and QFT+/TST+ results and between QFT+/TST- and QFT-/TST- was compared using Mann-Whitney test. We assessed the risk factors associated with TB infection using five different definitions of the LTBI status: (i) QFT+ vs. QFT-, (ii) TST+ vs. TST-, (iii) QFT+/TST+ vs. QFT-/TST-, (iv) discordant TST positive QFT-/TST+ vs. QFT-/TST- and (v) discordant QFT-GIT positive QFT+/TST- vs. QFT-/TST-. All analyses were conducted with a univariable and a multivariable mixed-effects logistic regression with a random effect per hospital to investigate the risk factors associated with LTBI. Statistical significance was considered to be a p-value under 0.05. All analyses were carried out using R software (version 3.4.0) and related packages “psych” and “lme4” [20–22].

## Results

### Healthcare workers

**Fig 1:**
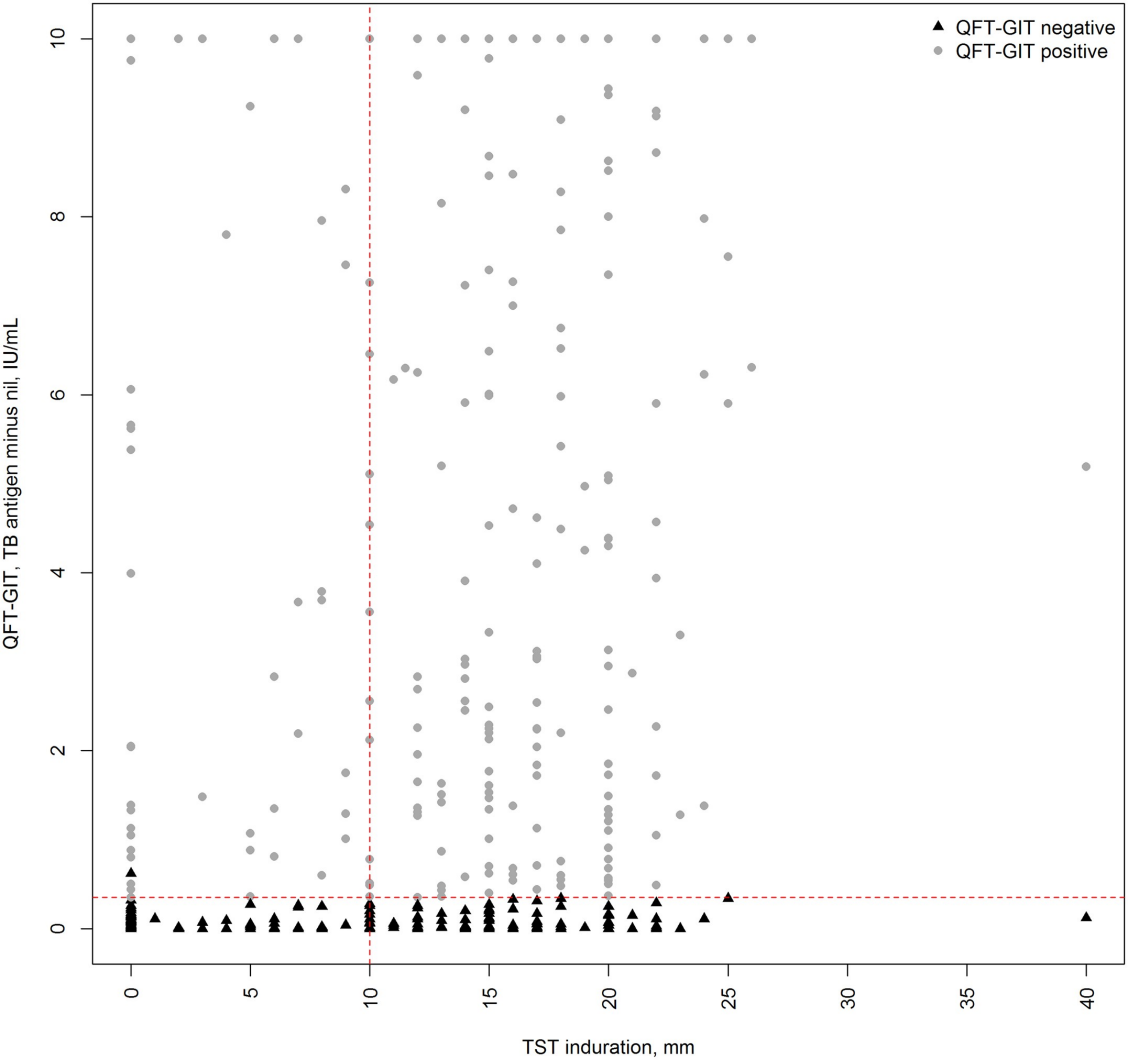
Scatterplot of quantitative QuantiFERON-TB Gold In-Tube (QFT-GIT) and tuberculin skin test (TST) results of 631 healthcare workers. The horizontal red dashed line represents a QFT-GIT cut-off of 0.35 IU/mL and the vertical one a TST cut-off of 10 mm. Samples with a QFT-GIT TB Antigen value inferior to the nil value were truncated to 0 (n = 122) and samples with a QFT-GIT TB Antigen value > 10 IU/mL were truncated to 10 (n = 63). One sample had a QFT-GIT TB Antigen minus nil value superior to 0.35 IU/mL but inferior to 25% of the nil value leading to a negative test result.

A total of 662 healthy HCWs were recruited from the hospitals of Rabat (n = 328) and Meknes (n = 334). We excluded 31 participants because of an indeterminate QFT-GIT result. Baseline characteristics of the 631 HCWs with reliable QFT-GIT results are displayed in Table 1. Overall, 54% of HCWs were male (n = 341), 47.1% were younger than 35 years old (n = 297) and 44.1% of them worked in a non-clinical department (n = 278). The majority of HCWs belonged to the medical staff (n = 321, 50.9%). The prevalence of QFT positivity was 40.7% (95%CI 36.9–44.5%) and that of TST positivity at the 10 mm cut-off was 52.1% (95%CI 48.2–56.0%) (Fig 1). When considering the LTBI status among individuals with concordant positive (QFT+/TST+) and concordant negative (QFT-/TST-) results, the prevalence of QFT+/TST+ was 45.2% (95%CI 40.7–49.5%).

**Table 1:** Baseline characteristics of 631 healthcare workers.

Characteristics	n (%)
Gender	
Male	341 (54.0)
Female	290 (46.0)
Age group (years)	
18–34	297 (47.1)
35–44	186 (29.5)
45–60	148 (23.4)
Comorbidities	
No	586 (92.9)
Yes	45 (7.1)
Smoking status	
No	549 (87.0)
Yes	82 (13.0)
Family history of TB	
No	588 (93.2)
Yes	43 (6.8)
Work area	
Non-clinical department	278 (44.0)
Surgical department	118 (18.7)
Clinical department	184 (29.2)
Pulmonology unit	51 (8.1)
Occupation	
Non-medical staff	167 (26.4)
Paramedical staff	143 (22.7)
Medical staff	321 (50.9)
QFT-GIT	
Negative	374 (59.3)
Positive	257 (40.7)
TST	
<10 mm	302 (47.9)
≥10 mm	329 (52.1)

QFT-GIT: QuantiFERON-TB Gold In-Tube; TB: tuberculosis; TST: tuberculin skin test

### Agreement between QFT-GIT and TST results

The agreement between QFT-GIT and TST results was slightly higher with a 10 mm TST cut-off [κ = 0.50; 95%CI 0.43–0.56; concordance of 74.6%] than with a 5 mm cut-off [κ = 0.48; 95%CI 0.41–0.54; concordance of 72.7%] or a 15 mm cut-off [κ = 0.44; 95%CI 0.37–0.51; concordance of 73.3%] (Table 2). Therefore, we kept the 10 mm cut-off for all further analyses on TST.

**Table 2:** Agreement between QFT-GIT and TST results in 631 healthcare workers according to various TST cut-offs (5 mm, 10 mm and 15 mm).

	TST 5 mm	TST 10 mm	TST 15 mm
QFT-/TST-	226	258	303
QFT+/TST+	233	213	160
QFT-/TST+	148	116	71
QFT+/TST-	24	44	97
Concordance, %	72.7	74.6	73.3
Κappa (95% CI)	0.48 (0.41–0.54)	0.50 (0.43–0.56)	0.44 (0.37–0.51)

QFT-GIT: QuantiFERON-TB Gold In-Tube; TST: tuberculin skin test

We further investigated the levels of the QFT-GIT and TST results in discordant and concordant subjects (S1 Table). Among 374 HCWs with QFT- results, the proportion of weak negative QFT-GIT result (0.2–0.34 IU/mL) was significantly higher (p = 0.0005) in discordant QFT-/TST+ subjects (17/116, 14.7%) than in double negative QFT-/TST- subjects (10/258, 3.9%). When focusing on 257 HCWs with QFT+ results, we did not observe any significant difference in the proportions of weak positive QFT-GIT result (0.35–0.7 IU/mL) between discordant QFT+/TST- subjects (5/44, 11.4%) and double positive QFT+/TST+ subjects (26/213, 12.2%). Among 302 HCWs with TST- results, the median induration size was significantly higher (p = 3.10^−9^) in discordant QFT+/TST- subjects (median = 3 mm) than in double negative QFT-/TST- subjects (median = 0 mm). Finally, among 329 HCWs with TST+ results, the median induration size was significantly lower (p = 0.0002) in discordant QFT-/TST+ subjects (median = 15 mm) than in double positive QFT+/TST+ subjects (median = 17 mm).

### Risk factors associated with LTBI status

When considering the LTBI status defined only by QFT-GIT results, univariable analyses showed that QFT+ was significantly associated with male gender (OR = 2.14; 95%CI 1.54–2.97, p = 6.10^−6^), older age between 35–44 years (OR = 1.80; 95%CI 1.23–2.63; p = 0.003) and 45–60 years (OR = 3.02; 95%CI 1.99–4.57; p = 2.10^−7^), smoking (OR = 2.57; 95%CI 1.59–4.15; p = 0.0001) and family history of TB (OR = 3.16; 95%CI 1.63–6.12; p = 0.0007). All these variables remained significant in multivariable analysis (Table 3).

**Table 3:** Risk factors associated with a positive QFT-GIT result in 631 healthcare workers.

Variables	Total n = 631	QFT- n = 374	QFT+ n = 257	OR_uni_^a^ (95%CI)	p_uni_^b^	OR_mult_^c^ (95%CI)	p_mult_^d^
Gender							
Female	290	200	90	Ref		Ref	
Male	341	174	167	**2.14 (1.54–2.97)**	**6.10**^**−6**^	**1.69 (1.17–2.46)**	**0.006**
Age group (years)							
18–34	297	207	90	Ref		Ref	
35–44	186	104	82	**1.80 (1.23–2.63)**	**0.003**	**1.57 (1.04–2.37)**	**0.03**
45–60	148	63	85	**3.02 (1.99–4.57)**	**2.10**^**−7**^	**2.74 (1.74–4.31)**	**1.10**^**−5**^
Comorbidities							
No	586	348	238	Ref		Ref	
Yes	45	26	19	1.08 (0.58–1.99)	0.82	0.95 (0.49–1.85)	0.88
Smoking status							
No	549	342	207	Ref		Ref	
Yes	82	32	50	**2.57 (1.59–4.15)**	**0.0001**	**1.89 (1.12–3.21)**	**0.02**
Family history of TB							
No	588	360	228	Ref		Ref	
Yes	43	14	29	**3.16 (1.63–6.12)**	**0.0007**	**3.41 (1.70–6.85)**	**0.0006**
Work area							
Non-clinical department	278	160	118	Ref		Ref	
Surgical department	118	79	39	0.94 (0.64–1.38)	0.74	1.40 (0.84–2.34)	0.19
Clinical department	184	106	78	0.68 (0.43–1.07)	0.10	0.73 (0.41–1.27)	0.26
Pulmonology unit	51	29	22	1.09 (0.59–2.01)	0.77	1.80 (0.88–3.69)	0.11
Job category							
Non-medical staff	167	89	78	Ref		Ref	
Paramedical staff	143	80	63	0.91 (0.58–1.43)	0.68	1.00 (0.58–1.74)	0.99
Medical staff	321	205	116	0.65 (0.44–1.05)	0.10	0.70 (0.41–1.19)	0.19

CI: confidence interval; QFT-GIT: QuantiFERON-TB Gold In-Tube; TB: tuberculosis

^a^OR_uni_: odds ratio obtained in a univariable regression

^b^p_uni_: p-value corresponding to the coefficient obtained in a univariable regression

^c^OR_mult_: odds ratio obtained in a multivariable regression including all the variables explored in univariable analyses

^d^p_mult_: p-value corresponding to the coefficient obtained in a multivariable regression including all the variables explored in univariable analyses

Bold text indicates a statistically significant result

When the LTBI status was defined only by TST, the same variables as those found in the previous analysis were significantly associated with TST+ in univariable analysis, with an additional one corresponding to working at a pulmonology unit (OR = 2.31; 95%CI 1.21–4.40; p = 0.01) (Table 4). In multivariable analysis, male gender (OR = 1.70; 95%CI 1.15–2.51; p = 0.008), older age between 35–44 years (OR = 2.80; 95%CI 1.82–4.30; p = 3.10^−6^) or 45–60 years (OR = 3.68; 95%CI 2.26–5.99; p = 2.10^−7^), family history of TB (OR = 4.89; 95%CI 2.04–11.74; p = 0.0004) and working at a pulmonology unit (OR = 3.79; 95%CI 1.77–8.13; p = 0.0006] were still associated with TST+. However smoking was no more a risk factor when adjusting for other covariates (OR = 1.27; 95%CI 0.71–2.26; p = 0.42) in particular gender, probably explained by the fact that all smokers were males (n = 82).

**Table 4:** Risk factors associated with a positive TST result in 631 healthcare workers.

Variables	Total n = 631	TST- n = 302	TST+ n = 329	OR_uni_^a^ (95%CI)	p_uni_^b^	OR_mult_^c^ (95%CI)	p_mult_^d^
Gender							
Female	290	165	125	Ref		Ref	
Male	341	137	204	**2.10 (1.50–2.94)**	**2.10**^**−5**^	**1.70 (1.15–2.51)**	**0.008**
Age group (years)							
18–34	297	185	112	Ref		Ref	
35–44	186	73	113	**2.58 (1.74–3.84)**	**3.10**^**−6**^	**2.80 (1.82–4.30)**	**3.10**^**−6**^
45–60	148	44	104	**3.50 (2.25–5.45)**	**3.10**^**−8**^	**3.68 (2.26–5.99)**	**2.10**^**−7**^
Comorbidities							
No	586	280	306	Ref		Ref	
Yes	45	32	23	0.98 (0.52–1.85)	0.95	0.82 (0.41–1.65)	0.58
Smoking status							
No	549	273	276	Ref		Ref	
Yes	82	29	53	**1.83 (1.10–3.05)**	**0.02**	1.27 (0.71–2.26)	0.42
Family history of TB							
No	588	294	294	Ref		Ref	
Yes	43	8	35	**4.04 (1.79–9.10)**	**0.0008**	**4.89 (2.04–11.74)**	**0.0004**
Work area							
Non-clinical department	278	144	134	Ref		Ref	
Surgical department	118	57	61	1.07 (0.72–1.59)	0.75	1.41 (0.83–2.41)	0.21
Clinical department	184	81	103	1.28 (0.81–2.02)	0.29	1.29 (0.73–2.29)	0.39
Pulmonology unit	51	20	31	**2.31 (1.21–4.40)**	**0.01**	**3.79 (1.77–8.13)**	**0.0006**
Job category							
Non-medical staff	143	60	83	Ref		Ref	
Paramedical staff	167	82	85	1.46 (0.91–2.35)	0.12	1.51 (0.84–2.71)	0.17
Medical staff	321	160	161	1.02 (0.69–1.51)	0.93	1.07 (0.61–1.88)	0.81

CI: confidence interval; TB: tuberculosis; TST: tuberculin skin test

^a^OR_uni_: odds ratio obtained in a univariable regression

^b^p_uni_: p-value corresponding to the coefficient obtained in a univariable regression

^c^OR_mult_: odds ratio obtained in a multivariable regression including all the variables explored in univariable analyses

^d^p_mult_: p-value corresponding to the coefficient obtained in a multivariable regression including all the variables explored in univariable analyses

Bold text indicates a statistically significant result

We then considered a more stringent definition of the LTBI status by focusing on the double positive QFT+/TST+ (n = 213) vs. the double negative QFT-/TST- subjects (n = 258). The same risk factors as those identified in the previous analysis focusing on TST only were found to be significantly associated with LTBI in a multivariable analysis (Table 5): being a male (OR = 2.21; 95%CI 1.40–3.49; p = 0.0007), having an older age between 35–44 years (OR = 2.43; 95%CI 1.45–4.06; p = 0.0007) or 45–60 years (OR = 4.81; 95%CI 2.72–8.52; p = 7.10^−8^), family history of TB (OR = 6.62; 95%CI 2.59–16.94; p = 8.10^−5^) and working at a pulmonology unit (OR = 3.64; 95%CI 1.44–9.23; p = 0.006). Again, smoking was not a significant risk factor for QFT+/TST+ when adjusting for other covariates (OR = 1.83; 95%CI 0.90–3.74; p = 0.10).

**Table 5:** Risk factors from multivariable regressions associated with positive concordant results (QFT+/TST+) results compared to the reference group (negative concordant results QFT-/TST-) in healthcare workers.

Variables	Total n = 471	QFT- TST- n = 258	QFT+ TST+ n = 213	OR_mult_^a^ (95%CI)	p_mult_^b^
Gender					
Female	229	152	77	Ref	
Male	242	106	136	**2.21 (1.40–3.49)**	**0.0007**
Age group (years)					
18–34	245	170	75	Ref	
35–44	121	56	65	**2.43 (1.45–4.06)**	**0.0007**
45–60	105	32	73	**4.81 (2.72–8.52)**	**7.10**^**−8**^
Comorbidities					
No	440	241	199	Ref	
Yes	31	17	14	0.80 (0.34–1.91)	0.62
Smoking status					
No	414	240	174	Ref	
Yes	57	18	39	1.83 (0.90–3.74)	0.10
Family history of TB					
No	436	251	185	Ref	
Yes	35	7	28	**6.62 (2.59–16.94)**	**8.10**^**−5**^
Work area					
Non-clinical department	204	115	89	Ref	
Surgical department	90	54	36	1.46 (0.78–2.72)	0.23
Clinical department	43	73	70	0.87 (0.44–1.72)	0.68
Pulmonology unit	34	16	18	**3.64 (1.44–9.23)**	**0.006**
Job category					
Non-medical staff	118	61	57	Ref	
Paramedical staff	107	52	55	1.31 (0.66–2.62)	0.44
Medical staff	246	145	101	0.82 (0.43–1.56)	0.54

CI: confidence interval; QFT: QuantiFERON-TB Gold In-Tube; TB: tuberculosis; TST: tuberculin skin test

^a^OR_mult_: odds ratio obtained in a multivariable regression including all the variables explored in univariable analyses

^b^p_mult_: p-value corresponding to the coefficient obtained in a multivariable regression including all the variables explored in univariable analyses

Bold text indicates a statistically significant result

### Risk factors associated with discordant QFT-GIT and TST results

Finally, we performed a specific analysis on the individuals with discordant results, QFT-/TST+ (n = 116) and QFT+/TST- (n = 44) that we compared to QFT-/TST- subjects (n = 258) (Table 6). For QFT-/TST+ individuals, we obtained in multivariable analysis results similar to those observed when considering all TST+ subjects with significant associations for male gender (OR = 1.73; 95% CI: 1.02–2.93; p = 0.04), older age between 35–44 years (OR = 4.18; 95%CI: 2.34–7.44; p = 1.10^−6^) or 45–60 years (OR = 4.35; 95%CI 2.16–8.75; p = 2.10^−7^) and working at a pulmonology unit (OR = 3.95; 95%CI 1.43–10.88; p = 0.008) without any effect of smoking (OR = 0.98; 95%CI 0.40–2.38; p = 0.97).

**Table 6:** Risk factors from multivariable regressions associated with discordant results (QFT-/TST+, QFT+/TST-) compared to the reference group (negative concordant results QFT-/TST-) in healthcare workers.

Variables	QFT- TST- n = 258	QFT- TST+ n = 116	OR_mult_^a^ (95%CI)	p_mult_^b^	QFT+ TST- n = 44	OR_mult_^a^ (95%CI)	p_mult_^b^
Gender							
Female	152	48	Ref		13	Ref	
Male	106	68	**1.73 (1.02–2.93)**	**0.04**	31	**2.37 (1.07–5.25)**	**0.03**
Age group (years)							
18–34	170	37	Ref		15	Ref	
35–44	56	48	**4.18 (2.34–7.44)**	**1.10**^**−6**^	17	2.41 (0.99–5.88)	0.05
45–60	32	31	**4.35 (2.16–8.75)**	**4.10**^**−5**^	12	**3.17 (1.18–8.50)**	**0.02**
Comorbidities							
No	241	107	Ref		39		
Yes	17	9	0.90 (0.35–2.36)	0.84	5	1.23 (0.35–4.40)	0.75
Smoking status							
No	240	102	Ref		33	Ref	
Yes	18	14	0.98 (0.40–2.38)	0.97	11	**3.35 (1.17–9.60)**	**0.02**
Family history of TB							
No	251	109	Ref		43	Ref	
Yes	7	7	1.89 (0.52–6.91)	0.33	1	1.65 (0.18–15.56)	0.66
Work area							
Non-clinical department	115	45	Ref		29	Ref	
Surgical department	54	25	1.27 (0.60–2.66)	0.53	3	0.80 (0.83–2.41)	0.68
Clinical department	73	33	1.22 (0.56–2.67)	0.62	8	**0.17 (0.04–0.72)**	**0.02**
Pulmonology unit	16	13	**3.95 (1.43–10.88)**	**0.008**	4	1.70 (0.43–6.72)	0.46
Job category							
Non-medical staff	61	28	Ref		21	Ref	
Paramedical staff	52	28	1.56 (0.69–3.55)	0.29	8	0.85 (0.29–2.53)	0.78
Medical staff	145	60	1.22 (0.55–2.68)	0.62	15	0.54 (0.20–1.49)	0.24

CI: confidence interval; QFT: QuantiFERON-TB Gold In-Tube; TB: tuberculosis; TST: tuberculin skin test

^a^OR_mult_: odds ratio obtained in a multivariable regression including all the variables explored in univariable analyses

^b^p_mult_: p-value corresponding to the coefficient obtained in a multivariable regression including all the variables explored in univariable analyses

Bold text indicates a statistically significant result

For QFT+/TST- individuals, we observed in multivariable analysis results close to those with the QFT+ analysis, with less significant p-values due to the smaller sample size. An association was observed with male gender (OR = 2.37; 95%CI 1.07–5.25; p = 0.03), older age which is borderline non-significant for the group 35–44 years (OR = 2.41; 95%CI 0.99–5.88; p = 0.05) and significant for the group 45–60 years (OR = 3.17; 95%CI 1.18–8.50; p = 0.02) and smoking (OR = 3.35; 95%CI 1.17–9.60; p = 0.02). We also noted a protective effect of working at a clinical department (OR = 0.17; 95%CI 0.04–0.72; p = 0.02).

## Discussion

We report here the first study of LTBI conducted in HCWs from Morocco, a middle-income country with intermediate endemicity for TB and high BCG vaccination coverage. The prevalence of LTBI ranged from 40.7% (36.9–44.5%) with QFT+ to 52.1% (48.2–56.0%) with TST+ and was 45.2% (40.7–49.5%) when considering the double positive QFT+/TST+ status. These values are consistent with prevalences observed in HCWs living in high TB burden countries such as Brazil (37%), China (54%), and India (43%) [6]. The higher proportion of TST+ compared to QFT+ in Morocco is consistent with a meta-analysis showing the same pattern in 24 out of 25 studies comparing IGRAs with TST in HCWs [23]. This observation may be explained in part by the higher specificity of IGRAs [9], and an extensive BCG vaccination coverage as it is the case in Morocco. The overall agreement between QFT-GIT and TST results (κ = 0.50 at a 10 mm cut-off) was also similar to previous estimates in high TB incidence countries (κ = 0.38 in a recent meta-analysis ranging from 0.19 in China to 0.61 in India [24]).

Interestingly, we identified risk factors that were consistently associated with LTBI, whatever its definition, namely gender, age, and familial history of TB. Male gender is a well-known risk factor for active TB disease due at least in part to socio-cultural causes as well as biological differences between sexes [25–26] but this gender disparity has not been fully investigated for LTBI. A significant higher proportion of LTBI in adult men than in adult women was reported in several studies [27–29] except one in a moderate TB burden country (Taiwan) that did not find gender as a risk factor while taking into account potential confounders [30]. The increase of LTBI prevalence with age is a well-known observation which has been previously reported either with QFT-GIT [31–32] or TST [33]. In our study, age was extremely correlated with the length of employment as almost all HCWs in military hospitals are recruited when they are young adults (between 18–21 years). Therefore, the increased risk of LTBI in the age groups belonging to 35–44 and 45–60 years is probably explained by a longer cumulative exposure that could be occupational and/or non-occupational.

We also identified other risk factors that reflect a close contact to infectious TB patients either through household exposure with a family TB history or an occupational one as working at a pulmonology unit, as already reported [27,31,34]. Beside the work area, we did not find any association with the job category and other potential occupational exposures may have not been captured. We also observed some interesting effects related to smoking. First, the smoking effect observed in univariable analyses decreased (QFT-GIT) or disappeared (TST) when adjusting for other covariates, in particular gender, probably because all smokers were males. In addition, when defining LTBI status by QFT-GIT, the smoking effect appeared to be stronger when considering discordant QFT+/TST- than concordant QFT+/TST+. Smoking was previously reported to be a risk factor for LTBI when assessed by QFT-GIT and/or TST [27–28,35], and also to affect the performance of the QFT-GIT test itself [36]. Further specific investigations should be conducted to investigate the relationship between smoking and IGRAs results with particular attention to confounding factors such as age, gender or socioeconomic status.

Finally, we focused on the discordant results between the two tests. As expected, QFT-/TST+ results were more frequent (116/631, 18.4%) than QFT+/TST- (44/631, 7.0%) [23]. The QFT-/TST+ discordance is often attributed in part to BCG vaccination and non-tuberculous mycobacteria. Interestingly, QFT-/TST+ subjects shared the same risk factors as those identified in the analysis for the QFT+/TST+ subjects. Moreover, we found a stronger proportion of weak negative QFT-GIT results in QFT-/TST+ than in QFT-/TST- (14.7% vs. 3.9%) indicating that some of these individuals may have a negative result due to the substantial variability of IGRA tests [10]. We also observed that the proportion of QFT-/TST+ was higher (79/334, 23.7%) in the oldest age group 35–60 years, when compared with the younger age group 18–34 (37/297, 12.5%) (S2 Table), consistent with the view that discordant QFT-/TST+ results may be due in part to temporally more remote infections [37]. Regarding the other discordant group QFT+/TST-, we also observed risk factors similar to those obtained with the QFT+ outcome, and this group had larger TST reactions than those of the QFT-/TST- group. Also, we cannot exclude that some of these QFT+/TST- subjects displayed a complete nonspecific cutaneous anergy that may explain a negative TST [38]. All these observations suggest that a substantial proportion of QFT+/TST- and QFT-/TST+ discordant HCWs may be truly infected individuals. Overall, the results we observed in terms of prevalence and risk factors for LTBI in HCWs living in Morocco were similar when using TST or QFT-GIT, suggesting that either could be used for a screening.

## Supporting information

S1 Table Comparison of discordant QFT-/TST+ vs. double negative QFT-/TST- results and QFT+/TST- vs. double positive QFT+/TST+ results according to QFT-GIT TB antigen minus nil categories. (DOC)

S2 Table Agreement between QFT-GIT and TST results in 631 healthcare workers according to age category and a TST cut-off at 10 mm. (DOC)
