# Supplementary material for: Prevalence and risk factors for latent tuberculosis infection among healthcare workers in Morocco
Source: PLoS One. 2019 Aug 15;14(8):e0221081. doi: 10.1371/journal.pone.0221081 (PMC6695119; doi:10.1371/journal.pone.0221081)
Supplement: S1 Table — (DOC) [file pone.0221081.s001.doc]

**S1 Table.** Comparison of discordant QFT-/TST+ vs. double negative QFT-/TST- results and QFT+/TST- vs. double positive QFT+/TST+ results according to QFT-GIT TB Antigen minus nil categories

|  | **QFT-/TST+**  **(n=116)** | **QFT-/TST-**  **(n=258)** | **p^a^** | **QFT+/TST-**  **(n=44)** | **QFT+/TST+**  **(n=213)** | **p^a^** |
| --- | --- | --- | --- | --- | --- | --- |
| TB Antigen minus nil (IU/mL) |  |  | 0.0005 |  |  | 1 |
| <0.2 | 99 | 247 |  | 0 | 0 |  |
| 0.2–0.34 | 17 | 10 |  | 0 | 0 |  |
| 0.35–0.7 | 0 | 1 |  | 5 | 26 |  |
| >0.7 | 0 | 0 |  | 39 | 187 |  |

QFT-GIT: QuantiFERON-TB Gold In-Tube ; TST: tuberculin skin test

^a^p-value of Fisher’s exact test
