# Supplementary material for: Prevalence and risk factors for latent tuberculosis infection among healthcare workers in Morocco
Source: PLoS One. 2019 Aug 15;14(8):e0221081. doi: 10.1371/journal.pone.0221081 (PMC6695119; doi:10.1371/journal.pone.0221081)
Supplement: S2 Table — (DOC) [file pone.0221081.s002.doc]

**S2 Table.** Agreement between QFT-GIT and TST results in 631 healthcare workers according to age category and a TST cut-off at 10 mm

|  | **TST 10 mm** |
| --- | --- |
| Age group 18-34, n=297 |  |
| QFT-/TST- | 170 |
| QFT+/TST+ | 75 |
| QFT-/TST+ | 37 |
| QFT+/TST- | 15 |
| Concordance, % | 82.5 |
| Κappa (95%CI) | 0.61 (0.52-0.71) |
| Age group 35-60, n=334 |  |
| QFT-/TST- | 88 |
| QFT+/TST+ | 138 |
| QFT-/TST+ | 79 |
| QFT+/TST- | 29 |
| Concordance, % | 67.7 |
| Κappa (95%CI) | 0.35 (0.26-0.45) |

QFT-GIT: QuantiFERON-TB Gold In-Tube ; TST: tuberculin skin test
